# The Position of Panel and Non-Panel Practitioners

**Published:** 1913-09-06

**Authors:** 


### THE POSITION OF PANEL AND NON-PANEL PRACTITIONERS.

### Three Classes of Panel Practitioners.

The classification of the men working the panel is not so easy because there is a different state of things existing in the provinces and in London, but in the main they fall into three classes, one of which needs subdivision, if it is to include both town and country. The first class that we catalogue are those men who from the first day of the introduction of the Insurance Act to the day on which it came into operation openly stated that they were enamoured of the new proposals and intended to work that Act whatever their professional brethren did. Those men were known and marked as enemies from the very start, and whilst one must deplore the attitude that they took up, yet one is bound to admit that they gave full notice of their intentions, and consequently are entitled to be treated as honourable opponents.

The second class contains men who at the time of the collection of the pledges by the British Medical Association willingly signed them, and on several occasions reiterated their intention of keeping to their pledged word, yet the ink was scarcely dry on their pledge form ere they again dipped the pen to sign the contract with the local Insurance Committee. The defection of these men at a critical moment in the campaign undoubtedly weakened the resolution of the loyal units and largely contributed to the melancholy spectacle of the members of a great profession eating their own words and chaining themselves to a bondage that they had so vigorously condemned.

The third class contains the great bulk of the profession, being made up of all those men who joined the panel posterior to the removal of the pledge, or joined on the understanding that the pledge was about to be rescinded. In many cases these men were "panicked" on by emissaries of the Government, by means over which it is best to draw a veil.

### How to Unite Under the One Flag.

In the first place, in any future defensive organisation of the profession all those individuals who occupy the first and second places in our classification of the panel men must be eliminated and reckoned as enemies pure and simple. This may seem a drastic method, but consider for a moment what would be the result of placing any further dependence on them. Assuming in some future conflict that they agreed to stand by the bulk of the profession, what reliance could be placed on their pledged word when they have so lightly broken it this time? Of what use would be their protestations of loyalty when so small a quantity of the Insurance Committee's gold tempted them this time? None whatever, and the sooner that the profession realises that in any future conflict they must reckon so many hundreds as against them so much the better for the profession. A patched-up truce that will break with the first sign of trouble is of no value and only gives a feeling of false security. So let us cut ourselves adrift from the class we mention and close up the ranks of those members that were loyal until release of the pledge or impending bankruptcy caused them to accept a servitude that they bitterly regret having taken.

Now as to the balance, who only need a proper lead to escape from the toils in which they labour. The interests of the unwilling panel practitioner and of the non-panel one are identical. Nothing will assist the non-panel men but an amendment of the measure that will ensure a liberal interpretation of the clause relating to contracting-out. This was the principle that actuated the London Medical Committee in their historical campaign of January last, and it is one of the foremost planks in the platform of the Guild. The attainment of the same is not so simple, but by organised effort much can and will be done. For example, at the present moment questions are being drafted that will be put to candidates for Parliament of all political classes and the profession will be instructed to sink their political opinions in favour of the candidate that embraces our views. This does not mean only to vote for them, but to use every means possible to get others to do so. The ballot-box is the only thing that any politician fears, and if we can strike them through their votes they will listen to reason. Very particular attention will be paid at the next General Election by the Guild to those constituencies where the majority is small either way, and all our powers will be thrown into this campaign.

